# Characterization of a Highly Toxigenic *Clostridium tetani* Strain from a Calf’s Castration Site

**DOI:** 10.3390/vetsci12100945

**Published:** 2025-09-30

**Authors:** Chie Shitada, Mayu Ohira, Mika Sekiguchi, Tomoko Koda, Motohide Takahashi, Makoto Kuroda

**Affiliations:** 1 Toxin and Biologicals Research Laboratory, Kumamoto Health Science University, Izumi-machi 325, Kita-ku, Kumamoto 861-5598, Japan; shitada@kumamoto-hsu.ac.jp; 2 The Chemo-Sero-Therapeutic Research Institute (KAKETSUKEN), Shinshigai 8-7, Chuou-ku, Kumamoto 860-0803, Japan; 3 Osaka Prefectural Livestock Hygiene Service Center, 1-59 Rinku Orai Kita, Izumisano 598-0048, Japan; 4 Graduate School of Veterinary Sciences, Osaka Metropolitan University, 1-58 Rinku-oraikita, Izumisano 598-8531, Japan; u21300b@omu.ac.jp; 5 Department of Medical Laboratory Science, Faculty of Health Sciences, Kumamoto Health Science University, Izumi-machi 325, Kita-ku, Kumamoto 861-5598, Japan

**Keywords:** calf, castration, tetanus, whole genome sequence, ELISA

## Abstract

**Simple Summary:**

We have isolated a high tetanus toxin-producing strain of *Clostridium tetani* from a calf. Due to their low sensitivity to tetanus toxin, calves may only develop tetanus when the bacteria produce exceptionally large amounts of toxin. If this hypothesis is correct, then strains with high toxin production would be selectively favored in low-susceptibility mammals such as calves and cattle, particularly if the disease were to become widespread. This has significant implications for human clinical settings as well. An increase in cases caused by these highly productive strains could diminish the efficacy of immunoglobulin antitoxin therapy.

**Abstract:**

Background: This case report describes a calf that underwent castration at a Japanese livestock farm and subsequently died after developing clinical signs of tetanus, including muscle rigidity and difficulty standing. Methods: A postmortem examination was performed, focusing on indurated lesions at the castration site, which were subjected to bacteriological and genetic analyses. Results: *Clostridium tetani* strain OPLHC-2022-Y645 was isolated from the purulent lesion. Whole-genome sequencing revealed a close genetic relationship to strain KHSU-254310-026, which belongs to the high toxin-producing lineage Clade 1-3 previously reported in Japan. Immunoassays demonstrated that OPLHC-2022-Y645 exhibited the highest tetanus toxin production among isolates tested to date. Conclusions: Whole-genome sequencing and immunoassay findings suggest that the rapid progression of tetanus in this calf could be associated with the strong toxin-producing capacity of the OPLHC-2022-Y645 strain.

## 1. Introduction

Tetanus is a wound infection caused by *Clostridium tetani*, an obligate anaerobic, spore-forming bacterium. In Japan, cattle, water buffalo, deer, and horses are designated as notifiable infectious diseases under the Domestic Animal Infectious Disease Control Law [1]. Among livestock, horses are the most susceptible, followed by guinea pig, sheep, mouse, goat, rabbit, dog and cat, whereas birds and cattle are generally resistant to tetanus [2]. Cattle are known to have unmeasurable low sensitivity to tetanus toxin [2], indicating that routine vaccination is not generally required because of the low sensitivity. Tetanus often arises after surgical procedures like childbirth, castration, and tail docking, as these wounds can become infected [3,4]. Umbilical cord infection is the primary cause in newborns [5]. *C. tetani* produces tetanospasmin, a potent neurotoxin that induces spastic paralysis by inhibiting the release of inhibitory neurotransmitters, leading to death in severe cases [6].

The number of reported tetanus cases in Japanese livestock varies by species: horses account for zero to several cases annually, while cattle account for approximately 70–100 cases per year [7]. Over the 10-year period from 2014 to 2024, the average annual number of reported cases in cattle was 98, whereas no horse cases have been reported since 2021 [7].

In addition, tetanus is classified as a Category 5 infectious disease under the Infectious Diseases Control Law in Japan. Veterinarians diagnosing tetanus in livestock and physicians diagnosing tetanus in humans are legally obligated to report cases to the relevant authorities. Globally, tetanus remains a major public health concern, particularly in developing regions with low vaccination coverage [8,9]. Prevention requires regular vaccination and proper wound management. Although tetanus toxoid vaccines are widely used in horses in Japan [10], cattle vaccination has not been fully established, which likely contributes to the continued reporting of tetanus cases in cattle [11]. While the risk of infection following surgical procedures such as castration is recognized, detailed reports of tetanus cases in cattle after castration remain scarce.

Recent research has shown that *C. tetani* strains vary considerably in their toxin-producing capacity [12]. Some high toxin-producing strains are suggested to cause more severe clinical symptoms [12], but the role of such strains in clinical cases has not been fully elucidated. This report describes the isolation of a high toxin-producing strain from a tetanus case that occurred after castration and examines its bacteriological characteristics and toxin-producing capacity.

## 2. Materials and Methods

### 2.1. Animal and Case History

A 6-month-old male F1 crossbred calf, born in March 2022, was castrated on a livestock farm in Japan. The calf had no history of tetanus vaccination. Open surgical castration was performed in September 2022. On day 7 post-castration, the animal began showing symptoms including bloating and teeth grinding. By day 8, severe bloating, hind limb extension, and difficulty standing were observed, and death was confirmed shortly thereafter.

### 2.2. Isolation of Clostridium tetani

The indurated lesions at the castration site were incised, and tissue fragments and exudate were collected and preserved in phosphate-buffered saline (PBS). Swab fluid and tissue fragments were each inoculated into cooked meat medium and cultured at 37 °C for 24 h. The obtained culture was inoculated onto sheep blood agar medium and subjected to anaerobic culture at 37 °C for 48 h, confirming bacterial motility on the medium. DNA was extracted from the motile colonies by alkaline heat treatment, and PCR analysis for tetanus toxin genes was performed. PCR used tetanus toxin gene-specific primers based on the report by Plourde-Owobi et al. (Forward: 5′-CTG GAT TGT TGG GTT GAT AAT G-3′ and Reverse: 5′-ATT TGT CCA TCC TTC ATC TGT AGG-3′) [13], using 25 pmol of each primer. Electrophoresis was performed on 2% agarose gel at 100 V for 30 min and stained with ethidium bromide for 10 min, confirming a band around 1354 bp.

### 2.3. Whole Genome Sequencing and Phylogenetic Analysis

Whole genome sequencing was performed using previously described methods [12]. The isolated strain was cultured in Brain Heart Infusion (BHI) medium at 37 °C for 24 h, then centrifuged at 8000 rpm for 20 min, and the recovered cells were inactivated by phenol-chloroform treatment. The bacterial suspension was disrupted using ZR BashingBead Lysis Tubes (Zymo Research, Irvine, CA, USA), and DNA was purified using MinElute PCR Purification Kit (QIAGEN, Hilden, Germany). DNA sequencing libraries were prepared using QIAseq FX (QIAGEN) and sequenced using NextSeq 2000 sequencer (Illumina, San Diego, CA, USA) with 150 bp paired-end reads. The obtained reads were used to construct a draft genome through de novo assembly using SPAdes v3.15.2, and genome annotation was performed using DFAST [14].

Furthermore, core genome phylogenetic trees and pairwise single nucleotide variants (SNVs) were determined using Parsnp v1.7.4 [15]. Phylogenetic relationships were evaluated using iQtree version 2.2.5 [16] with 1000-fold bootstrapping using the following parameters (iqtree2-s parsnp.snps.mblocks-alrt 1000-B 1000-nt 6). Phylogenetic tree was visualized by iTOL web site (https://itol.embl.de/ accessed on 19 August 2025) [17].

### 2.4. Immuno-Assay Using Enzyme-Linked Immunosorbent Assay (ELISA)

Tetanus toxin detection was performed according to the method of Shitada et al. [12]. The isolated strain was cultured in BHI medium at 37 °C for 4 days. The culture supernatant after centrifugation at 8000 rpm for 20 min was used as the sample. This was then added to plates immobilized with anti-tetanus rabbit polyclonal antibodies and reacted with horseradish peroxidase-labeled anti-tetanus mouse monoclonal antibodies. After 3,3′,5,5′-tetramethylbenzidine (SeraCare Life Sciences, Milford, MA, USA) addition, the reaction was stopped and absorbance at 450/620 nm was measured. The toxin from the isolated strain was determined as a relative value using parallel line assay with a standard strain (KHSU154301-001) as reference.

## 3. Results

### 3.1. Animal Case History and Isolation of C. tetani

**Figure 1:**
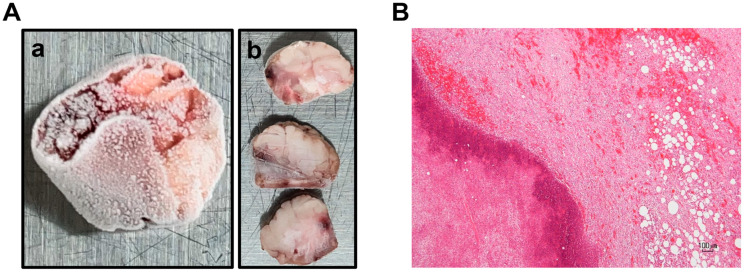
Gross and histological appearance of indurated lesions formed at castration site. (**A**) a: Tissue fragment collected from indurated lesions formed at the castration site of the deceased calf. b: Cut surface of the tissue fragment. (**B**) Abscess. Granulation tissue formation and collagen fiber proliferation surrounding the pus. Hematoxylin and eosin staining. Scale bar: 100 μm.

Postmortem examination revealed indurated lesions containing pus approximately 7.0 cm in diameter at the castration site. Histologically, abscesses with surrounding collagen fiber proliferation were observed (Figure 1). Isolation of *C. tetani* OPLHC-2022-Y645 was described in detail in Section 2.2.

### 3.2. Whole Genome Sequencing

**Figure 2:**
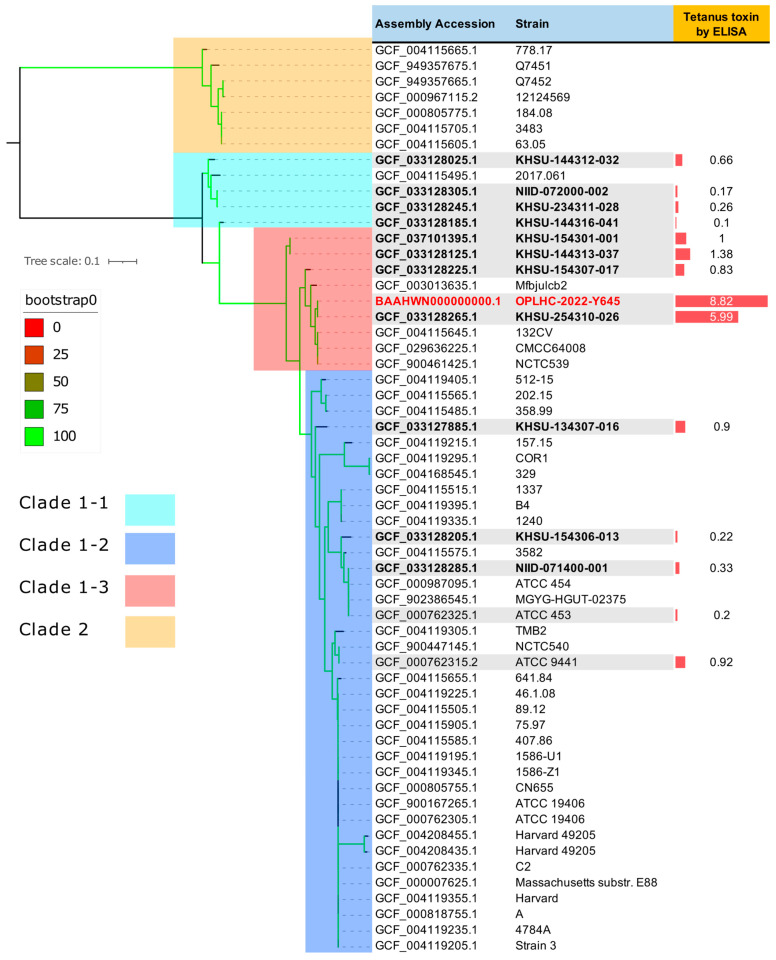
Core-genome SNVs phylogenetic analysis of *C. tetani* OPLHC-2022-Y645 strain.

Whole genome sequencing and subsequent core-genome phylogenetic analysis suggested that OPLHC-2022-Y645 is closely related to KHSU-254310-026, which belongs to the high toxin-producing lineage of Clade 1-3 previously reported in Japan (Figure 2) [12].

A maximum-likelihood phylogenetic analysis was performed using 57 representative *C. tetani* strains. Their whole-genome sequences were retrieved from the NCBI Datasets database (https://www.ncbi.nlm.nih.gov/datasets/genome/?taxon=1513, accessed on 20 February 2024). The reference genome used was *C. tetani* E88. The tree scale bar indicates the substitution rate, and bootstrap values (%) with 1000 replicates are shown as indicated by the color on the branches. Phylogenetic clades were highlighted with a colored background. Tetanus neurotoxin expression was determined by ELISA for only available strains as a relative value using a parallel line assay. The OPLHC-2022-Y645 strain produced 8.6-fold more toxin than the standard reference strain (KHSU154301-001). For most strains, only genomic information can be downloaded, so it was not possible to measure toxin production levels in the isolates.

### 3.3. Detection of Tetanus Toxin by ELISA

**Figure 3:**
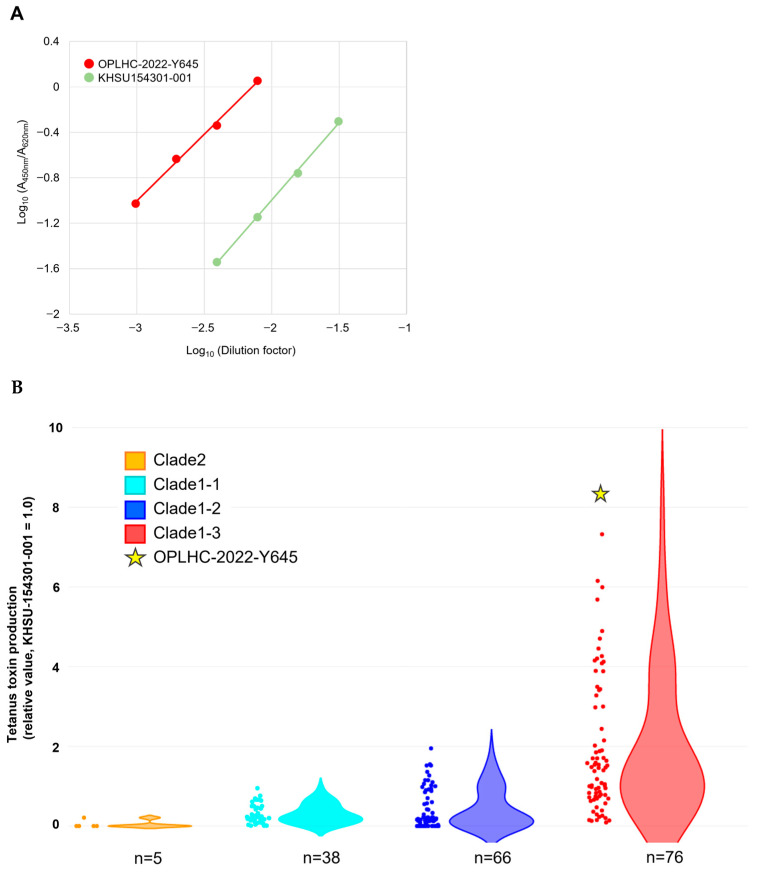
Tetanus toxin production of OPLHC-2022-Y645. (**A**) Detection of tetanus toxin by sandwich ELISA. Compared to the standard strain KHSU154301-001, OPLHC-2022-Y645 showed approximately an 8-fold higher toxin production. (**B**) Comparative analysis of tetanus neurotoxin expression in culture supernatants among genomic clades. Original expression data, which were previously reported [12], were re-evaluated with the expression of OPLHC-2022-Y645 (highlighted with a yellow star) from the current study.

Tetanus neurotoxin expression was quantified by ELISA as a relative value using a parallel line assay with the standard strain KHSU-154301-001 as the reference (Figure 3A) [12]. The strain OPLHC-2022-Y645 produced approximately eight times more toxin than the reference strain, identifying it as a high-level toxin-producing strain (Figure 3B). Among the strains tested, OPLHC-2022-Y645 showed the highest expression value (8.82-fold; 95%CI: 7.65–10.28) (Figure 2 and Figure 3B).

## 4. Discussion

Generally, clinical isolation of *C. tetani* has been challenging, and tetanus is typically diagnosed on the basis of clinical symptoms in hospital. The taxonomic identity and genetic characteristics of this organism have been elucidated primarily through genomic analyses and reports of previously isolated strains [18,19], including those obtained from environmental soil in Kumamoto Prefecture, Japan [12]. In the present study, we conduct a comparative genomic analysis of animal-derived strain—particularly calf isolate, which remains exceedingly rare—against previously characterized genomes (Figure 2). To the best of our knowledge, this is the first whole-genome sequence of *C. tetani* isolated from a calf. Furthermore, quantitative assessment of toxin production levels was performed to evaluate potential variations in virulence.

In this case, wound infection at the castration site likely caused tetanus in the calf, suggesting the infection progressed rapidly after the surgical procedure. While clinical symptoms and bacterial culture results are important diagnostic clues, we confirmed that tetanus toxin detection is essential for a definitive diagnosis.

The high toxin-producing capacity of the isolated OPLHC-2022-Y645 strain is considered a contributing factor to the rapid progression of tetanus. This strain, which produces approximately 8 times more toxin than the standard strain (Figure 3), suggests that both the quantity and quality of the toxin significantly influence disease severity.

Recent research has shown that the toxin-producing capacity of *C. tetani* varies markedly among strains. Some strains belonging to Clade 1-3, isolated in Japan, have been reported to have extremely high toxin-producing capacity [12]. Our case strain was also found to belong to Clade 1-3, suggesting that *C. tetani* isolated from clinical specimens may have very high toxin-producing capacity. The presence of these high-toxin-producing strains is likely a key factor in the onset and rapid progression of tetanus, implying that specific lineages may be involved in disease severity.

In Japan, tetanus vaccines are widely used for horses [11], but vaccination is not widespread in cattle. Horses typically have established tetanus vaccination programs, including a two-dose primary immunization administered four weeks apart, followed by boosters every six to twelve months. In contrast, cattle do not routinely receive tetanus vaccination as a standard preventive measure, which is considered a factor contributing to the higher incidence of tetanus in cattle compared to horses. The incidence of bovine tetanus remains low, with approximately 100 cases reported annually in Japan, even in the absence of a comprehensive vaccination program. For economic reasons, vaccinating all cattle is considered unfeasible, and consequently, vaccination of cows is also limited. No treatment is generally administered for cattle, as therapeutic effects are not expected. Ursodeoxycholic acid injection, Lestion V (Methionine + vitamins, etc.: nutritional support, adjunctive therapy for poisoning) and fluid replacement solutions (Ringer’s solution, glucose solution, etc., for purposes such as improving dehydration) may be prescribed, but this is at the veterinarian’s discretion. Future recommendations should include tetanus vaccination for cattle to prevent the disease; otherwise, as Kim et al. report, establishing a treatment regimen that ensures appropriate and timely care will be necessary [20].

Furthermore, thorough wound management, sterilization of instruments, and ensuring a clean surgical environment are crucial. Contaminated instruments and inappropriate surgical environments increase the risk of infection, so meticulous hygiene is required during surgical procedures. This is expected to enable more effective prevention of infections such as tetanus.

## 5. Conclusions

Calves that show muscle rigidity after castration should be strongly suspected of having tetanus, requiring early diagnosis and appropriate treatment. In this case, symptoms progressed rapidly, leading to the calf’s death. Cattle are known to have low sensitivity to tetanus toxin [2], which suggests that the disease may only develop in these animals when the bacteria produce large amounts of toxin. This might present one of the concerns with the effectiveness of immunoglobulin antitoxin therapy. Since this study is based on only a single case, additional investigation is warranted to fully characterize the high-toxin-producing lineage.

When treating suspected tetanus cases, it is crucial to consider the potential presence of high-toxin-producing strains during both diagnosis and treatment. To avoid such outcomes, thorough vaccination and other preventive measures, including surgical techniques that prevent bacterial infection, are essential. Specifically, when performing surgical procedures like cattle castration, it is vital to take appropriate measures to mitigate the risk of *C. tetani* infection.

## Data Availability

The draft genome sequence data of OPLHC-2022-Y645 in this study has been deposited in the DNA Data Bank of Japan (DDBJ) and is publicly available under accession numbers BAAHWN010000001-BAAHWN010000046 (46 contigs) and BioSample: SAMD01593429.
